# Intravitreal Ranibizumab Alone or in Combination with Calcium Dobesilate for the Treatment of Diabetic Macular Edema in Nonproliferative Diabetic Retinopathy Patients: 12-Month Outcomes of a Retrospective Study

**DOI:** 10.1155/2022/6725225

**Published:** 2022-10-20

**Authors:** Dongxuan Wang, Hui Wang, Shuang Wu, Xueqiu Yang, Jiansen Xu

**Affiliations:** Department of Ophthalmology, Changyi People's Hospital, Changyi, Weifang 261399, Shandong, China

## Abstract

**Objective:**

This study investigates the efficacy of CaD combined with intravitreal ranibizumab for the treatment of diabetic macular edema (DME) in patients with nonproliferative DR.

**Methods:**

This retrospective, observational, case-control study enrolled consecutive patients newly diagnosed with DME. The patients were treated with 3-monthly loading dose injections of intravitreal ranibizumab (IVR) followed by pro re nata injections (3 + PRN), with or without daily oral CaD. The patients were treated and followed up for 12 months. We reviewed their medical records to determine the optical coherence tomography (OCT) findings, number of injections, best-corrected visual acuity (BCVA), and central macular thickness (CMT) at 3, 6, and 12 months after the first injection.

**Results:**

We reviewed 102 eyes of 102 patients; 54 patients received IVR combined with oral CaD (IVR + CaD group) and 48 received only IVR (IVR group). In both groups, BCVA was higher, and CMT was lower, at 3, 6, and 12 months after the injection compared to those at the baseline (*p* < 0.05 for all), while there were no significant differences in BCVA improvement or CMT reduction between the two groups (*p* > 0.05). The mean number of IVR injections was significantly lower in the IVR + CaD group than the IVR group (5.4 ± 1.1 vs. 6.7 ± 1.6 injections, *p* < 0.05) during 1 year of treatment. No adverse events were noted in either group.

**Conclusions:**

Compared to IVR alone, the addition of oral CaD to IVR in DME patients was safe and effective for improving visual function and restoring the retinal anatomy and was associated with the need for fewer injections.

## 1. Introduction

Diabetic macular edema (DME) is the leading cause of visual impairment in patients with diabetes mellitus and is characterized by exudative fluid accumulation in the macula [1, 2]. The blood-retinal barrier (BRB) is vital for maintaining normal structure and function of the retina. DME is caused by damage to the BRB [3]. BRB disruption is associated with increased production of the vascular endothelial growth factor (VEGF), intercellular adhesion molecule-1, interleukin-6, monocyte chemotactic protein-1, and other inflammatory factors [3]. Therefore, anti-VEGF [4, 5] and anti-inflammatory agents [6] are the main treatment methods for DME after laser photocoagulation. Although the Diabetic Retinopathy Clinical Research Network randomized clinical trial [7] reported that intravitreal aflibercept, bevacizumab, and ranibizumab injections improved vision in eyes with center-involved DME (CIDME), a significant number of patients have a poor or short-duration response to anti-VEGF treatment. Although anti-VEGF therapy is a cost-effective way to improve vision loss in DME [8], frequent intravitreal injections impose a high economic burden on patients, particularly in developing countries. Therefore, studies have investigated combination therapy using intravitreal anti-VEGF agents with intravitreal triamcinolone acetonide [9], Rho kinase inhibitors [10], methotrexate [11], subthreshold micropulse lasers [12, 13], macular grid laser photocoagulation [14], and subtenon steroid injections [15], as well as switching from anti-VEGF treatment to intravitreal dexamethasone implantation [6, 16–18], for DME patients.

Calcium dobesilate (calcium 2, 5-dihydroxybenzenesulfonate; CaD) is a vascular-protective drug used for the treatment of diabetic retinopathy (DR) [19–21] due to its ability to prevent oxidative stress and inflammation [22]. It protects blood vessels and improves the circulation by lowering blood viscosity, inhibiting platelet activity, and reducing capillary permeability [23, 24]. A previous randomized, double-blind, placebo-controlled, multicenter clinical trial reported that CaD did not reduce the risk of development of DME [25]. However, the potential beneficial effects of combined use of CaD and intravitreal anti-VEGF agents on DME patients are not clear. Therefore, we investigated the effectiveness and safety of combination therapy with CaD and anti-VEGF agents for the treatment of DME in nonproliferative DR patients. The primary outcomes of this study were the change in best-corrected visual acuity (BCVA) and central macular thickness (CMT) after treatment. The secondary outcome was the number of intravitreal ranibizumab (IVR) injections administered during the study period.

## 2. Materials and Methods

### 2.1. Study Design and Participants

**Figure 1:**
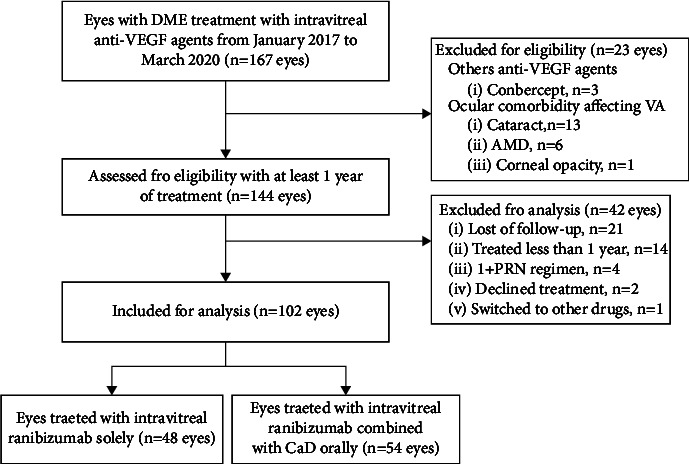
Flowchart of patient selection.

This retrospective study enrolled treatment-naive patients with nonproliferative DR and CIDME, who received IVR at the Department of Ophthalmology, Changyi People's Hospital. The patients were followed up for 1 year. All patients had type 2 diabetes mellitus and clinically significant DME. According to the ETDRS criteria [26], clinically significant macular edema is defined as retinal thickening that involves or threatens the center of the macula (even if visual acuity is not yet reduced).  Figure 1 presents the flowchart of patient selection.

This study adhered to the Declaration of Helsinki and was approved by the Ethics Committee of Changyi People's Hospital. Informed consent was obtained from the patients or their guardians before IVR. The medical records were retrospectively reviewed after approval was received from the Institutional Review Board of Changyi People's Hospital.

### 2.2. Data Collection

The data retrieved from the medical records included demographic information, medical history, history of ocular conditions, and previous treatments for DME. The participants underwent detailed ophthalmological examination, including BCVA, slit-lamp biomicroscopy, intraocular pressure measurement, fundus examination under pupil dilation, and macular scans using spectral domain optical coherence tomography (OCT; Cirrus HD-OCT [software version 6.0]; Carl Zeiss Meditec, Dublin, CA, USA), before treatment and at each follow-up. The CMT was defined as the retinal thickness of the central 1.0 mm, as determined using an OCT B-scan. Complications during the perioperative and postoperative period were also recorded.

### 2.3. Treatment Protocol

A 3-monthly loading dose of IVR, followed by pro re nata injections (3 + PRN), was used for anti-VEGF treatment of patients. The criteria for reinjection were similar to those described previously [27]. At each visit, patients underwent visual acuity assessment and OCT to determine the macular thickness. If BCVA and OCT were stable over two consecutive visits, and reinjection was suspended; reinjection was considered in cases showing deterioration of BCVA (loss of >5 letters) or OCT (CMT increase by > 10%) at follow-up.

In the IVR group, patients received IVR injections only, while in the IVR + CaD group, patients received oral CaD (1,500 mg per day in three 500 mg doses) and IVR injections.

### 2.4. Statistical Analysis

The data were analyzed using SPSS software (version 20.0; IBM Corp., Armonk, NY, USA). The BCVA measurements were converted to logMAR equivalents for statistical analysis. The Pearson chi-square test was used for comparative analyses of categorical variables. The independent sample *t*-test and paired *t*-test were performed to analyze changes in BCVA and CMT. *P* < 0.05 was considered statistically significant.

## 3. Results

Between January 2017 and March 2020, 102 patients (102 eyes; mean age: 57.72 ± 9.91 years; range: 42–69 years) were enrolled in this study, of whom 63 (61.76%) were males and 39 (38.24%) were females. The IVR + CaD and IVR groups included 54 and 48 patients, respectively.

Table 1 presents the baseline characteristics of the study participants. There were no significant differences between the groups in sex, age, smoking, alcohol use, or comorbidities, except in terms of the presence, treatment course, and control (i.e., HbA1c) of diabetes.

After 3-monthly loading doses of IVR, BCVA (logMAR) significantly increased from 0.35 ± 0.12 to 0.24 ± 0.09 (*p* < 0.05) in the IVR group and from 0.32 ± 0.15 to 0.23 ± 0.10 in (*p* < 0.05) in the IVR + CaD group (Table 2). The BCVA remained stable in both treatment groups until the end of the follow-up (Table 2). Furthermore, the CMT significantly decreased from 427 ± 172 to 286 ± 69 *μ*m (*p* < 0.05) in the IVR group and decreased from 412 ± 185 to 277 ± 92 *μ*m (*p* < 0.05) in the IVR + CaD group. The CMT remained stable in both treatment groups until the end of the follow-up. No significant difference was observed in BCVA or CMT between the two groups at the baseline or follow-up visits.

After 1 year of IVR treatment, the mean number of injections was 6.7 ± 1.6 in the IVR group, which was significantly greater than that in the IVR + CaD group (5.4 ± 1.1, *p* < 0.05). There were no ocular complications (such as endophthalmitis, vitreous hemorrhage, and retinal detachment) or serious systemic adverse effects (such as cerebral or myocardial infarction) in either group.

## 4. Discussion

DME is a type of diabetic maculopathy and important cause of vision loss among individuals with diabetes worldwide [2]. The advent of anti-VEGF therapy has significantly improved the outcomes of DME patients, and intravitreal anti-VEGF injections are considered the first-line therapy for DME. However, some patients respond poorly to treatment.

CaD is a vascular-protective drug with beneficial effects for vascular diseases, such as chronic venous insufficiency [28], hemorrhoids [25], DR [29–31], and multiple microangiopathic diseases [32]. Previous studies have suggested that several mechanisms underlie the improvement in microcirculation and reduction in microvascular injury associated with CaD use [21]. First, CaD reduces platelet aggregation caused by thrombin or collagen [33]. Second, it significantly protects the peritoneal vessels from the penetrative effects of reactive oxygen species [34]. Third, it inhibits capillary permeability [35]. Fourth, it alleviates chronic inflammation and improves endothelial cell function [36, 37]. Fifth, it reduces endothelial shedding by increasing the synthesis and release of nitric oxide [38]. Sixth, it inhibits prostaglandin production to reduce platelet aggregation, erythrocyte aggregation, and suspension viscosity [24]. Seventh, it downregulates the expression of the VEGF and fibroblast growth factor to inhibit vascular endothelial cell proliferation [39]. Finally, it restores autophagy by inhibiting the VEGF/PI3K/AKT/mTOR signaling pathway [40]. Therefore, CaD acts through several mechanisms and is effective in the treatment of DME. However, in a previous study, CaD did not reduce the risk of development of DME [25]. Furthermore, in another study, CaD combined with laser photocoagulation did not decrease macular thickness in DME patients compared to placebo [41]. Both of the aforementioned studies were published before the anti-VEGF agents became popular. No previous studies have investigated the effectiveness and safety of oral CaD combined with anti-VEGF treatment. Therefore, our study is the first to demonstrate that, and compared to anti-VEGF monotherapy, CaD combined with anti-VEGF agents had similar effects in terms of improving visual function and reducing CMT in DME patients, and it reduced the number of intravitreal injections and economic burden during the 1-year follow-up period.

Although the exact mechanism underlying the effects of DME is still unclear, abnormally increased levels of VEGF and inflammatory mediators, and subsequent breakdown of BRB, play a vital role in the development of DME [2]. Accordingly, anti-VEGF and anti-inflammatory agents are considered the first-line treatments for DME [42, 43] and show good outcomes [44, 45]. CaD downregulates VEGF and inhibits VEGF-related pathways and may work synergistically with anti-VEGF agents [46]. This might partly explain why oral CaD alone has no therapeutic effect on DME, but it reduced the number of intravitreal injections required when combined with anti-VEGF agents during the 1-year follow-up period of this study. In addition, the treatment combination was safe, with no systemic or local complications observed. Although CaD inhibited VEGF expression by acting synergistically with anti-VEGF agents and prolonging their effect duration, it did not increase the risk of adverse events due to VEGF inhibition.

This study has some limitations. First, it was a retrospective study with a relatively small sample size, which may have made it difficult to detect small but significant changes in visual and retinal structure improvement. Secondly, the follow-up period was relatively short, and this may not have allowed for enough time to detect the efficacy and safety of CaD during long-term treatment. Thirdly, this study only included type 2 diabetes DME patients in the NPDR stage, and the results could not give more information to type 1 diabetes DME patients or DME patients in the PDR stage.

In summary, oral CaD combined with IVR has similar effectiveness and safety as anti-VEGF monotherapy for improving visual function and restoring the retinal anatomy in DME patients and reduced the need for IVR injections. The addition of oral CaD can reduce the number of IVR injections required and the economic burden of treatment in DME patients receiving anti-VEGF therapy.

### 4.1. A Statement for Preprint

There is a preprint version of the manuscript as shown by the following link: https://assets.researchsquare.com/files/rs-830956/v1/d501c711-99ff-4725-ab9e-d2180e8fe71a.pdf?c=1634186654 [47].

## Figures and Tables

**Table 1:** Baseline characteristics of total study population.

	IVR (*n* = 48)	IVR + CaD (*n* = 54)	*p* value
Gender (male/female)	31/17	32/22	0.581
Age (years)	56.98 ± 11.62	57.65 ± 12.79	0.784
Smoking (yes/no)	28/20	29/25	0.638
Drinking (yes/no)	19/29	25/29	0.494
Comorbidity (yes/no)	17/31	18/36	0.825
Course (years)	10.58 ± 6.00	11.51 ± 6.16	0.440
HbA1c (%)	7.72 ± 0.88	7.80 ± 1.01	0.645

**Table 2:** Changes of CMT and BCVA after the treatment.

	BCVA (logMAR)			CMT (*μ*m)		
	IVR (*n* = 48)	IVR + CaD (*n* = 54)	*p* value	IVR (*n* = 48)	IVR + CaD (*n* = 54)	*p* value
Baseline	0.35 ± 0.04	0.34 ± 0.04	0.416	428.21 ± 100.68	424.48 ± 88.42	0.843
At 3 month	0.24 ± 0.04^^*∗*^^	0.23 ± 0.04^*∗*^	0.253	288.27 ± 19.83^*∗*^	282.06 ± 26.55^*∗*^	0.188
At 6 month	0.26 ± 0.04^*∗*^	0.25 ± 0.04^*∗*^	0.436	281.40 ± 27.34^*∗*^	274.33 ± 31.74^*∗*^	0.234
At 12 month	0.25 ± 0.03^*∗*^	0.24 ± 0.04^*∗*^	0.696	269.33 ± 28.82^*∗*^	264.93 ± 27.16^*∗*^	0.429

^*∗*^represents *p* < 0.05 when compared with the corresponding parameters at the baseline.

## Data Availability

The data that support the findings of this study are available on request from the corresponding author. The data are not publicly available because of privacy or ethical restrictions.

## Additional Points

What is already known about this topic? CaD is a vascular-protective drug used for the treatment of DR. It protects blood vessels and improves the circulation by lowering blood viscosity, inhibiting platelet activity, and reducing capillary permeability. Although a previous study reported that CaD did not reduce the risk of development of DME, the potential beneficial effects of the combined use of CaD and intravitreal anti-VEGF agents on DME patients are not clear. What does this article add? Within one year of IVR treatment by 3 + PRN regimen, adding oral CaD to intravitreal ranibizumab might be a feasible way to reduce the injection number and decrease the economic burden of treatment in DME patients who received anti-VEGF therapy.
